# The Effect of Social Media Use on Depressive Symptoms in Older Adults with Self-Reported Hearing Impairment: An Empirical Study

**DOI:** 10.3390/healthcare9111403

**Published:** 2021-10-20

**Authors:** Yiming Ma, Changyong Liang, Xuejie Yang, Haitao Zhang, Shuping Zhao, Liyan Lu

**Affiliations:** 1 The School of Management, Hefei University of Technology, Hefei 230009, China; mayiming923@163.com (Y.M.); xuejie_y@126.com (X.Y.); zhaoshuping1753@hfut.edu.cn (S.Z.); ly18255173836@163.com (L.L.); 2 The School of Management, Jilin University, Changchun 130012, China; zhtinfo@126.com

**Keywords:** hearing impairment, social relationship, S-O-R, depressive symptoms, subjective aging, social media

## Abstract

Older people with hearing impairment are more likely to develop depressive symptoms due to physical disability and loss of social communication. This study investigated the effects of social media on social relations, subjective aging, and depressive symptoms in these older adults based on the stimulus-organism-response (S-O-R) framework. It provides new empirical evidence to support improving the mental health and rebuilding the social relations of older people. A formal questionnaire was designed using the Wenjuanxing platform and distributed online through WeChat; 643 valid questionnaires were received from older people with self-reported hearing impairments, and SmartPLS 3.28 was used to analyze the data. The results show that (1) social media significantly impacts the social relations of older people with hearing impairment (social networks, β = 0.132, T = 3.444; social support, β = 0.129, T = 2.95; social isolation, β = 0.107, T = 2.505). (2) For these older people, social isolation has the biggest impact on their psychosocial loss (β = 0.456, T = 10.458), followed by the impact of social support (β = 0.103, T = 2.014); a hypothesis about social network size was not confirmed (β = 0.007, T = 0.182). Both social media (β = 0.096, T = 2.249) and social support (β = 0.174, T = 4.434) significantly affect the self-efficacy of hearing-impaired older people. (3) Both subjective aging (psychosocial loss, β = 0.260, T = 6.036; self-efficacy, β = 0.106, T = 3.15) and social isolation (β = 0.268, T = 6.307) significantly affect depressive symptoms in older people with hearing impairment. This study expands the theories of social media aging cognition, social support, and social networks and can provide practical contributions to the social media use and mental health of special persons 60 years and older.

## 1. Introduction

At present, the percentage of people over 60 years old in the world has exceeded 14% [1], and about one-third of these older adults are troubled by deafness or hearing loss [2]. Most studies have shown a significant association between hearing impairment and depression in older adults. It is common practice to improve the hearing level of older individuals by using hearing aids or cochlear implants. However, suffering hearing loss has a serious negative impact on their social relations, and it is often difficult for these older adults to rebuild their social relations afterward. With the popularity of social media, more and more older people use social media actively or passively [3,4]. Various communication methods (video, audio, picture, expression, etc.) provided by social media can improve the decline of communication ability caused by aging [5]. Compared with non-users of social media, older adults who use social media have a lower amount of clinical depression [6].

Existing studies have explored this group as they search online for health information [7], the improvement of their cognitive ability [5], the elimination of hearing impairment [8], and other aspects. These studies show the positive impact of social media use on older adults with hearing impairment. However, there are few empirical studies on the relationship between social media use and depressive symptoms in older adults with hearing impairment. Most studies on hearing impairment and depression in the older people continue to focus on the mechanisms of their onset, including social isolation [9], cognitive ability [10], loneliness [11], etc. The effect of increased social relationships caused by social media use on improved depressive symptoms in the current ICT environment was not considered. In addition, although some studies have discussed the influence of social media use on the social interaction of older adults [12], different studies consider only some dimensions of social interaction, such as social support [13], the social network [14], and the formation of loneliness [3]. There is a lack of an integrated perspective to study the impact of different dimensions of social interaction on depression in older adults. Finally, in contrast with depressive symptoms in other age groups, depressive symptoms in older adults are often the result of the combined effects of physical, psychological, and social aging [15]. In particular, the cognition of aging in psychological and social aspects has an important impact on older adults [16]. However, current research related to social media and aging also focuses on cognitive improvement [17], learning ability [18], and executive function [5], and lacks research on how social media can improve aging perception in older adults [19]. Therefore, existing studies lack sufficient empirical evidence on the relationship between social media and individual cognition of aging.

To fill the above research gaps, this paper constructs an integrated research model based on the stimulus-organism-response (S-O-R) theory to explore the influence mechanism of social media use on the subjective aging and depressive symptoms of hearing-impaired older people. The S-O-R framework, first proposed by Mehrabian and Russell (1974), states that a stimulus (S) received by individuals from the environment triggers assessments of their internal states (organism, O), resulting in positive or negative responses (R) [20]. The framework believes that the cues (stimuli) perceived from the environment can trigger a person’s internal assessment state (organisms), and then produce a psychological and cognitive state response to the stimulus. This can reflect a series of changes in the individual, caused by external influences, well. Existing studies have confirmed that the S-O-R framework has a good effect on the analysis of the formation of individual psychological problems. Pandita, Mishra, and Chib, for example, used this framework to study the psychological effects of COVID-19 on individuals [21]. Yang et al. studied the effects of metacognitive beliefs and catastrophic misconceptions on health anxiety in social media use [22]. Cao et al. also used the S-O-R model to investigate the effect of social media use on fatigue and on dependence on internalization disorders (depression and anxiety) [23]. Therefore, the S-O-R framework can effectively measure mental health changes caused by external stimuli. In addition, social-media-related research has also adopted the S-O-R framework. Cao and Sun (2018) explored the impact of overload on discontinuous intentions of social media users from the perspective of S-O-R [24]. Liu (2021) used the S-O-R model to study the use of social media during the COVID-19 pandemic [25]. Whelan, Islam, and Brooks (2020) studied the relationship between social media overload and fatigue using a stress–strain results approach [26]. Therefore, compared with other theories, the S-O-R framework provides a reasonable explanation for the impact of social media on the mental health of older people with hearing impairment.

Therefore, this research unifies the concepts of aging cognition, social relations, and psychology to establish a conceptual model of S-O-R that reflects the whole. That is, the changes in social relationships (stimuli) that are caused by using social media can cause changes in the subjective aging cognition of older people with hearing impairment (organisms) and exert a positive or negative influence on their depressive symptoms (responses). It will be helpful to reveal social media’s influence mechanism on the mental health of hearing-impaired older people from a process perspective. Based on the S-O-R framework, this study constructs a theoretical model of the impact of social media use on depressive symptoms in older people with hearing impairment. Stimulus (S) consists of social media use and social relations. Social relations are divided into social isolation, social network scale, and social support. Organism (O) shows the influence of social media use and social relationship on the subjective senility of the older individuals. It includes the following two dimensions: social psychological loss and self-efficacy. We took depressive symptoms as a response (R) to explore the effects of perceived aging and social isolation on the mental health of older people with hearing impairment.

In conclusion, the main purpose of this study is to reveal the effects of social media usage on aging cognition and the mental health of older people with self-reported hearing impairment. In addition, the influence of social media usage on the social isolation and subjective aging of older people was also analyzed. Based on the research’s purpose and objective, questionnaires were designed online using the Wenjuanxing Questionnaire Platform, and then distributed to older Chinese people by using WeChat. The study uses the structural equation model analysis method, uses SmartPLS software to analyze the collected data, and finally, draws research conclusions.

The rest of the study is organized as follows: Section 2 introduces the theoretical basis of the study and develops the hypotheses. Section 3 describes the scale and data collection methods. Section 4 describes the results of the data analysis. Finally, the results and findings are discussed, and practical contributions are discussed.

## 2. Literature Review and Hypothesis Development

### 2.1. Social Relationship

Social relationship is a multi-dimensional concept, including various structures, functions, and qualities [27]. The structure of social relationship refers to the individuals with whom one has an interpersonal relationship and the linkages between these individuals [28], that is, the social network composed of people. Members of a social network include relatives, friends, colleagues, and neighbors. The function of social relations is defined as interpersonal interaction within the structure of those social relations [28]. Through interpersonal interaction, individuals can obtain various types of resources and support, namely, social support [27]. The quality of social relations reflects the actual contact or relationship level between an individual and society, such as the level of social participation, social isolation, relationship tension, etc. [16].

As the social relations of older adults are not a uniform whole, the roles of different dimensions of these social relations can be quite different [27]. Older people with different characteristics have different social relationship needs. For example, older people usually rely on their social network to obtain relevant social support, but older adults with independent living ability or better economic status may have lower social support needs [29]. Older people who are sociable tend to try to maximize the size or quality of their social networks [30]. For older adults with hearing impairment, the scale of their social network may be small due to communication disorders, and they may lose the ability to live independently to a certain extent [31]. In addition, in terms of the quality of social relations, the decrease in social interaction leads to a greater possibility of social isolation [32]. Therefore, in this study, we take social network size, social support, and social isolation as three dimensions to measure social relations.

### 2.2. Subjective Aging

Aging is a complex concept, including biological (physical), psychological, and social aging [33]. Physical aging refers to the gradual loss of physiological integrity, resulting in impaired function and an increased risk of death [34]. Psychological and social aging refer to the changes in individual psychological status and social relations, respectively, as age increases [35]. Subjective aging refers to individuals’ views on their own aging [36]. Individuals develop their senescence cognition by sensing changes in their biological, social, and psychological functions [37]. An individual’s assessment of his or her age may deviate from his or her actual age [37]. Physical aging is often inevitable, but individual psychological and social aging can be improved through psychological and behavioral resources, social support, social communication, social and cultural atmosphere, etc. [38]. Therefore, it is important to improve the subjective aging of older adults and to help them realize active aging by improving their psychological and social cognition of aging.

For psychological and social aging, there are differences in the selection of constructs according to different research focuses. Social aging cognition is often measured in terms of social identity, respect, social relationships, and work-related losses [15,39]. Psychological adjustment, self-efficacy, psychological stress, and psychological resources have been used to measure the cognition of psychological aging [37,40,41]. The research object of this paper is older people with hearing impairment. The older adults in this group are often troubled by the decline in their communication abilities, and the loss of interpersonal relationships will be more prominent. Therefore, loss of social relations was chosen as a construct to measure social aging. For older adults, psychological aging is reflected in the loss of control beliefs, resulting in low mobility or depressive symptoms [27]. In addition, because of physical health problems, control beliefs are more important in older adults with hearing impairment, to adjust the relationship between mental and physical. As an effective psychological control resource, self-efficacy plays an important role in maintaining physical and mental health [42]. Therefore, self-efficacy was used as a construct to assess psychological aging in this study.

### 2.3. Hypothesis Development

#### 2.3.1. Influence of Social Media Use

For older adults with hearing impairment, social media offers a new way to communicate [7]. Social media has rich means of expression, such as communicating through pictures, words, videos, and emojis, which can make up for the communication barriers caused by hearing loss. This new way of communicating has a positive impact on all dimensions of social relations among the older adults in this group. As mentioned above, we believe that social media can affect the structure (social network size), function (social support), and quality (social isolation) of social relationships in older adults with hearing impairment.

Social network size is defined as the number of individuals or organizations that an individual can simultaneously monitor [43]. As social networking is the most important function of social media, social media often directly affects the social network size of older adults. Through social media, older adults can more easily contact their family and friends and maintain the existing scale of their social network [44]. Additionally, with the help of social media, older adults can reconnect with friends and relatives who are not often contacted due to space limitations and restore their original social network. In addition, social media features such as community and connectivity can help older adults expand their social networks. By joining various social circles (such as WeChat groups) where relatives and friends are active, older adults can make new friends [44]. Posting public information on social media can attract responses from others, which can also expand the social networks of older adults [45]. Therefore, this study assumes the following:

**Hypothesis** **1.** **(H1).** *Social media use positively affects social network size*.

Social support is defined as the help, care, or resources that an individual receives [46]. Hearing loss can lead to a decline in older adults’ ability to obtain social support and can affect their quality of life [47]. Through social media, older adults can access more social support. When older adults begin to learn how to use social media, they can often obtain social support from their children, relatives, or friends to help them learn how to use it [48]. When they master the methods of using social media, they can effectively obtain social support from their own social networks [49]. With the in-depth use of social media, older adults can obtain various types of social support from online channels. For example, when visiting an online health community to discuss health issues, they can obtain information support, emotional support, peer support, etc. Therefore, this study assumes the following:

**Hypothesis** **2.** **(H2).** *Social media use will positively affect the level of social support*.

Social isolation refers to an insufficient quality and quantity of social interaction between individuals and other people, groups, and communities [50]. For older people with hearing impairment, the scale of their social networks and the amount of social support they receive are often not enough to be substantive social interaction. On the one hand, affected by hearing loss, older adults may not be able to communicate effectively with others. Although they may receive care or social support (i.e., more attention from surrounding individuals or organizations), the actual number and quality of their social interactions are low [51]. On the other hand, the physical disability caused by hearing impairment may make them think that they are incomplete, which may cause them to have low self-esteem or other negative emotions [52]. Although hearing impairment can be improved by using hearing aids, it may still lead them to actively reduce their face-to-face social interaction [52]. Social media can alleviate the social anxiety that comes from face-to-face communication, which can promote people’s social interaction online. With the help of social media, older adults tend to be more willing to communicate with members of their social network [53]. Moreover, the group characteristics of social media can help people find groups of others in the same situation as themselves, which means individuals in the community have a more common language, improving the frequency and quality of social interaction, and reducing the sense of social isolation [54]. In conclusion, we believe that social media can improve the sense of social isolation of older people with hearing impairment. Therefore, this study assumes the following:

**Hypothesis** **3.** **(H3).** *Social media use will positively affect the sense of social isolation*.

#### 2.3.2. Subjective Aging

From the perspective of social factors, the loneliness caused by the decrease in social relations is an important factor affecting the cognition of aging [55]. The loss of contact with colleagues due to retirement and the reduction in social network size due to the death of social network members and/or the onset of physical disability are important causes of loneliness in older adults [55,56]. Especially for older people with hearing impairment, communication disorder is an important cause of a reduction in social network size [57]. In addition, a decrease in the amount of social support also affects the subjective aging of older adults [58]. Affected by the reduction in social network size, the overall amount of social support of older adults will significantly decrease, which will affect their cognition of aging. Since most of the social support for older adults comes from family members, when the family members do not live in the same city or when a partner dies, it significantly impacts their social support [56]. This can also lead to increased feelings of aging. Finally, influenced by multiple factors, older adults often lack interaction with other age groups [59]. This often makes them feel socially alienated and socially withdrawn, resulting in a lack of social participation and them feeling abandoned by the times [59]. Moreover, for older people with hearing impairment, hearing loss will make it difficult for them to interact or communicate with others [60]. This often makes it difficult for them to integrate into society and deepen their understanding of aging. In conclusion, we believe that all dimensions of social relationships have a significant impact on the cognition of social aging in older adults with hearing impairment. Therefore, this study assumes the following:

**Hypothesis** **4.** **(H4).** *The scale of the social network affects the cognition of social aging*.

**Hypothesis** **5.** **(H5).** *The level of social support affects the cognition of social aging*.

**Hypothesis** **6.** **(H6).** *Social isolation affects the perception of social aging*.

Self-efficacy is defined as an individual’s subjective judgment on whether he or she can successfully perform a certain achievement behavior [61]. Self-efficacy is influenced by various factors such as past success or failure experiences, indirect experiences, internal interest, and emotion [62]. As a kind of efficacy expectation, the self-efficacy level of older adults is often derived from the stereotype of aging and is strengthened through the individual’s positive and negative experiences. Therefore, self-efficacy is an important index to predict individual subjective aging [37]. For older adults, social media can effectively improve their sense of self-efficacy. Learning and successfully using social media can increase self-efficacy in older adults [63]. Participation in and use of social media also increases their sense of self-efficacy [64]. Moreover, more social support can be obtained through social media. Different dimensions of social support can positively affect the self-efficacy of older individuals. For example, emotional support can provide positive emotions in daily life and improve self-confidence. Information support can provide all kinds of information resources that older adults need to provide them with indirect experience. Tool support can provide practical help for older adults to improve their executive function. In conclusion, we believe that social media and social support can improve the self-efficacy of older adults with hearing impairment. Therefore, this study assumes the following:

**Hypothesis** **7.** **(H7).** *Social media use affects self-efficacy*.

**Hypothesis** **8.** **(H8).** *The level of social support affects self-efficacy*.

#### 2.3.3. Depressive Symptoms

Although the occurrence of depressive symptoms is influenced by many factors, subjective senescence is often an important cause of depressive symptoms in older individuals [65]. How an individual perceives aging may influence how they respond to life events or diseases [66]. When older adults experience the inconveniences of aging, weak control (i.e., poor self-efficacy) can make them feel more stressed [67]. Older people with a younger subjective age tend to have a more positive outlook on life, greater self-confidence, and a lower likelihood of depressive symptoms [66]. Psychosocial loss plays an important role in the depressive symptoms of older adults [68]. Psychosocial aging will reduce older adults’ desire to participate in society, actively or passively reducing their interaction and contact with others, which often leads to loneliness and depressive symptoms in the older individuals. Loneliness tends to further deepen older adults’ perception of social aging, creating a vicious circle [59]. In addition, social isolation is an important predictor of depressive symptoms in old age [69]. Social isolation not only leads to psychosocial loss in older adults, but also directly affects the generation of depressive symptoms in this population. Older people affected by social isolation tend to have negative physiological and psychological reactions such as cognitive impairment, decreased sleep quality, and anxiety, and are more likely to have depressive symptoms [70]. In particular, for older people with hearing impairment, social isolation, while passive, has an important effect on the development of depressive symptoms. In conclusion, we believe that subjective aging (psychosocial loss and self-efficacy) and social isolation will have a significant impact on depressive symptoms in older adults with hearing impairment. Therefore, this study assumes the following:

**Hypothesis** **9.** **(H9).** *Psychosocial loss can significantly affect depressive symptoms*.

**Hypothesis** **10.** **(H10).** *Self-efficacy can significantly affect depressive symptoms*.

**Hypothesis** **11.** **(H11).** *Social isolation can significantly affect depressive symptoms*.

**Figure 1:**
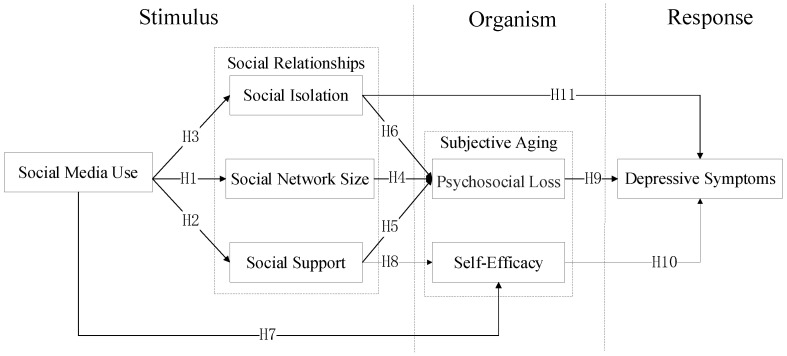
Research model.

Based on the S-O-R theory, we built a model for social media use on depressive symptoms in older people with hearing impairments. In this model, social media use and social relationships together constitute stimuli. Social media use also affects social support, social network size, and social isolation for older people. We use subjective aging as organism, including self-efficacy and psychosocial loss. All the dimensions of social relations (social support, social network size, and social isolation) have an impact on the psychosocial loss of older persons. Social media use and social support can have an impact on the self-efficacy of hearing-impaired older people. Depressive symptoms are the ultimate response of individuals, affected by social isolation and subjective aging. The research model is shown in Figure 1.

## 3. Research Methodology

### 3.1. Measurement

Based on the previous literature review and hypothesis development, our proposed theoretical model includes seven latent variables, including social media use, social network size, social support, social isolation, psychosocial loss, self-efficacy, and depressive symptoms. Specifically, the measures of social media use were adapted from Boer et al. (2021) [71]; the measures of social network size were adapted from Kuiper et al. (2020) [72]; the measures of social support were adapted from Zhang (2017) [73]; the measures of social isolation were adapted from Nicholson (2020) [74]; the measures of psychosocial loss were adapted from related questions from the Attitudes to Ageing Questionnaire (AAQ) [75]; the measures of self-efficacy were adapted from the General Self-Efficacy Scale (GSE) [76]; and the measures of depressive symptoms were adapted from the Geriatric Depression Scale 5 (GDS-5) [77]. All questionnaires had Chinese translations and were proven to have good reliability and validity in previous studies. All items adopted a seven-point Likert scale where 1 represents negative (strongly disagree or strongly agree) and 7 represents positive (strongly agree or strongly disagree), with the exception of social media use (SMU1, where 1 represents less than 1 year, to 7, more than 7 years; SMU2, where 1 represents never or less than once, to 7, more than 40 h; SMU3, where 1 represents less than once, to 7, more than 80 times; and SMU4, where 1 represents less than once, to 7, more than 20 times). The contents of the questionnaire are shown in Table 1.

### 3.2. Ethical Consideration

This study was approved by the Academic Committee of Hefei University of Technology. The questionnaire had a detailed informed consent form before it is completed and could only be completed if the respondents chose to agree. Anonymity and confidentiality were guaranteed, and the study did not collect personal information such as respondents’ names, cell phone numbers, addresses, etc.

### 3.3. Eligibility Criteria

#### 3.3.1. Inclusion Criteria

Inclusion criteria for participants in our research are as follows:

- Have experience in using social media and are using social media;
- Are over 60 years old;
- Have self-reported hearing impairment.

#### 3.3.2. Exclusion Criteria

Exclusion criteria for participants in our research are as follows:

- Have dual sensory loss (hearing and vision).

### 3.4. Data Collection and Sample

This study collected firsthand data by means of a questionnaire survey and conducted empirical analysis. There were several steps to the data collection. In order to ensure the quality of the questionnaires, 50 copies of a pre-survey questionnaire were distributed offline. All 50 were collected. Some of the respondents were interviewed, and the questionnaire was revised according to their suggestions.

The formal questionnaire was designed using the WenJuanXing platform and distributed online through WeChat (convenience sampling). In order to prevent repeated filling of the questionnaire, we set up access rules to ensure that the same IP address or WeChat account could only fill out the questionnaire once. We set some invalid questionnaire rules in the questionnaire. A questionnaire with no self-reported hearing impairment, with all the same answers, or with inconsistent forward and reverse answers was excluded. The questionnaires were collected in July 2021, and the respondents were people over 60 years old who use social media (WeChat). A total of 1827 questionnaires were collected. After eliminating the invalid questionnaires, 643 valid questionnaires were received, with an effective rate of 35.19%.

According to the rules we set, the questionnaire had to be completed before all items could be submitted. Therefore, in this study, all the questions in the questionnaire were filled in and there were no missing values. In order to reduce the cognitive burden on older persons in the process of answering, in the demographic information part, we set the fixed options (no manual filling); in the formal question, we used the Likert scale of seven grades, with values ranging from 1 to 7. All questions were descriptions of the subjective feelings of older people, and there was no right or wrong answer. Therefore, there is no problem with outliers in this study. The sample characteristics of the questionnaire are shown in Table 2.

Table 3 shows the mean and standard deviation (SD) of the model. According to the analysis results, the mean value range of each variable item of the model is from 4.751 to 5.056, and the standard deviation range is from 1.476 to 1.680, indicating that the data are relatively concentrated, with little fluctuation, and have good adaptability.

## 4. Results

### 4.1. Measurement Model Testing

The main analysis method of this paper is partial least squares (PLS). According to Hair (2012), in exploratory research, PLS is more suitable than other methods [78]. In addition, PLS has relatively loose requirements on the normal distribution of sample data and has high flexibility when processing data that are missing values. Therefore, this paper uses SmartPLS 3.2.8 (Boenningstedt, Germany) for data analysis.

#### 4.1.1. Common Method Bias and Multicollinearity

The variance inflation factor (VIF) is measured. VIF can measure the severity of collinearity in multiple linear regression models. The value of VIF is greater than one, and the closer its value is to one, the lighter the multicollinearity is. When the VIF value is less than five, it indicates that there is no multicollinearity problem in the model [78]. In this study, the maximum value of VIF is 1.938, which is less than the threshold value five, indicating that the model does not have a multicollinearity problem.

Harman’s one-factor test was used to identify any potential common method bias [79]. If the single factor accounts for more than 50% of the variance, it indicates that the questionnaire data may have the possibility of common method bias [80]. Principal component factor analysis showed that the first five factors accounted for 57.827% of the total variance, and the percentage of the first (largest) factor was 19.585%, with no factor higher than 50%. Therefore, the possibility of common method deviation in this study is small.

#### 4.1.2. Reliability and Validity

Table 4 shows the factor loading, Cronbach’s alpha, rho_A, combined reliability (CR), and extracted mean variance (AVE). According to the suggestion of Hair (1998), the factor loading of the model should be at least 0.60, and ideally, it should be 0.70 or higher. In this study, the model’s factor loading ranges from 0.703 to 0.859, which is greater than the ideal value of 0.7 [78]. This indicates that the observed variables have strong convergent validity, and that there is a high correlation between the observed variables and their structural variables. CR is an important index to measure the internal reliability of each dimension of the model, which should be greater than 0.7 [78]. In this study, the value range of CR was 0.865–0.916, which was greater than the threshold of 0.7. It shows that the model has good convergent validity. Cronbach’s alpha is an important index to measure the internal validity of the model, and it is recommended that it be greater than 0.7 in most of the literature [78]. In this study, the value range is 0.765–0.900, which is larger than the threshold value of 0.7, indicating that the questionnaire has good internal consistency. The value of the Cronbach coefficient may underestimate the actual reliability; therefore, Dijkstra and Henseler suggested rho_A for supplementary analysis [81]. According to Henseler et al. (2016), the rho_A value should be greater than 0.7 [81]. In this study, the value range of rho_A is 0.770 to 0.922, which is larger than the threshold value of 0.7, indicating that the model construction has good reliability. In addition, the AVE values range from 0.523 to 0.680, all of which are greater than 0.5, indicating that the observed items explain the difference much more than the error term, and the effectiveness of model aggregation is relatively high [78].

Table 5 shows the discriminant validity. The square roots of all the AVEs are greater than the following, indicating that the questionnaire questions have good discriminant validity [82].

### 4.2. Structural Model

**Figure 2:**
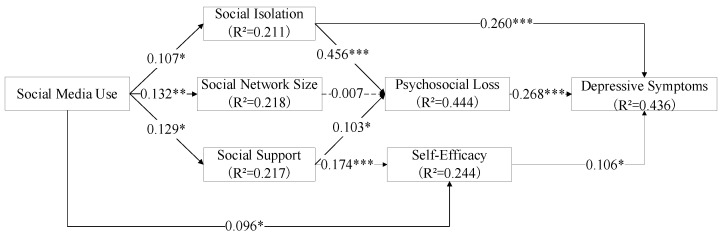
Model results. *** *p* < 0.001; ** *p* < 0.01; * *p* < 0.05.

In this study, we used SmartPLS 3.28 for data analysis. Bootstrapping was adopted, and the maximum number of iterations was 3000. The specific results are shown in Table 6 and Figure 2.

As shown in Table 6, only hypothesis H4 is not supported, and the rest of the hypotheses are supported.

Finally, we conducted a control variable test. The *t*-test results show that sex, age, and income have no significant impact on this research. The results show that demographic characteristics have no significant influence on the results of the analysis.

## 5. Discussion

### 5.1. Findings

The purpose of this study was to reveal the association between social media use and depressive symptoms as self-reported in older people with hearing impairment. Based on our research objectives, under the S-O-R framework, we constructed a model of the impact of social media use on depressive symptoms in older people with hearing impairment. Through the testing of the model hypotheses, we reached the following conclusions:

(1) Social media has a significant impact on the social relations of hearing-impaired older adults, and can improve the social network size, social isolation, and social support in their social relations. However, in terms of specific impact, social media has the greatest impact on the scale of social networks (H1, path coefficient = 0.132, T = 3.444, *p* = 0.001); second is the impact on social support (H2, path coefficient = 0.129, T = 2.95, *p* = 0.003); and third is social isolation (H3, path coefficient = 0.107, T = 2.505, *p* = 0.013). This shows the characteristics of social media use among older people with hearing impairment.

Consistent with previous opinion, social media use can effectively improve the social relationship structure of older adults with hearing impairment and help them communicate with more groups or individuals [44]. This result shows the positive significance of social media for older adults with hearing impairment. Comparing the structure and function of social relations shows that the influence of social media on social support is smaller than that on the scale or size of the social network. It shows that older people are more likely to use social media to seek support from a strong offline network of relationships, especially when they encounter difficulties in daily life. Cantor (1979) pointed out that older adults want to receive social support first from their spouses; then from their children, relatives, and friends; and finally, from professionals or formal organizations [83]. As an early study, the social environment was significantly different from the current one; however, considering the current older population, the formation of their ideas is consistent with the previous study. This reflects that the current older population is not deeply affected by the social media environment, as there has been no significant change in thinking. This is a valuable discovery, which can provide new support for the theory of social networks and social support of older adults in the social media environment.

In addition, social media use also has a positive impact on the social isolation of hearing-impaired older adults. Social media can increase the level of individual social interaction [12]. Social media is mostly used for booking offline social activities, while online social interaction is often ignored [84]. For older people with hearing impairment, social media can provide some social interaction, but the more focused offline social interaction is still affected by physical disability. This may explain why social media has a lower impact on social isolation. The findings help expand the existing literature on the effects of social isolation on specific groups.

(2) Different dimensions of social relationships have different effects on the senescence cognition of hearing-impaired older adults. For older people with hearing impairment, social isolation has the biggest impact on their psychosocial loss (H6, path coefficient = 0.456, T = 10.458, *p* < 0.001), followed by the impact of social support (H5, path coefficient = 0.103, T = 2.014, *p* = 0.044), and the hypothesis of social network size is not established (H4, path coefficient = 0.007, T = 0.182, *p* = 0.856).

The findings reveal attitudes and perceptions about social media use and social relationships among older groups. The results of a study conducted by Bell et al. (2013) showed that many older adults do use Facebook, but their main purpose is to stay in touch with family [85]. This is probably the main reason why hypothesis four does not work. That is, social media use by older adults is aimed at connecting them with strong social relationships, rather than expanding their social networks. Although social media has expanded the scale of their social networks, they still care most about their relatives or close friends, and their main social support comes from strong social relations [12]. Considering the significant correlation between social isolation and psychosocial loss in older persons, we suggest that the significant impact on social isolation in older persons comes from their family and friends [55]. When older adults have less social interaction with their family or friends, they experience strong social isolation and suffer psychosocial losses. This shows the main source of psychosocial loss suffered by older individuals. The social connections obtained from social media can improve this problem. This is a valuable finding that contributes to the development of cognitive theories of aging in older adults and could explain the inconsistent results of previous studies on social media use by older individuals. In addition, the findings also contribute to the development and application of positive aging theory.

Both social media and social support have significant effects on the self-efficacy of hearing-impaired older individuals. Self-efficacy is affected by many factors, but the degree of influence of different factors on individual self-efficacy is also different [62,86]. Compared with the influence of social media (H7, path coefficient = 0.096, T = 2.249, *p* = 0.025), social support has a greater impact on the self-efficacy of older people with hearing impairment (H8, path coefficient = 0.174, T = 4.434, *p* < 0.001). Although the successful learning and use of social media by older adults can promote the improvement of self-efficacy [63], consistent with previous studies, the self-efficacy of older adults is mainly derived from social support [64]. Different dimensions of social support (tool support, emotional support, information support, and economic support) can provide comprehensive help for the improvement of the self-efficacy of older individuals. Although social media can also provide some emotional and informational help, there is a gap in quality and quantity compared with social support. Social media serves more as an effective communication tool to facilitate older adults’ access to social support. This result shows the status and role of social media in promoting self-efficacy in older individuals. The findings contribute to the development of theories related to social support and self-efficacy in the context of social media.

(3) Both subjective aging and social isolation have significant effects on depressive symptoms in older people with hearing impairment. Compared with self-efficacy (H10, path coefficient = 0.106, T = 3.15, *p* = 0.002), social isolation (H11, path coefficient = 0.268, T = 6.307, *p* < 0.001) and psychosocial loss (H9, path coefficient = 0.260, T = 6.036, *p* < 0.001) related to social connections had a greater impact on depressive symptoms among older people with hearing impairment [87]. However, this is a gradual process, and these older people tend to come to terms with their diminished self-control [88]. For individuals, the main impact of hearing loss is a reduction in the individual’s ability to communicate. As a result, the older adults in this group often need more care and communication from others. However, as hearing loss worsens, the frequency and number of social interactions declines more significantly, leading to a greater desire for social engagement. Therefore, factors related to social participation will have a greater impact on their mental health level [89]. This may be the reason why the effect of social isolation and psychosocial loss on depressive symptoms in older people with hearing impairment is greater than that of self-efficacy. This is an important research finding that has important theoretical significance for studying the needs cognition of older adult groups with different characteristics. It also provides new empirical evidence and directions for improving the mental health of certain groups of older people.

### 5.2. Implications for Practice

This research has the following practical implications:

(1) Although many older people are using social media, their main purpose in doing so is to build strong relationships in their in-person social network, and the depth and breadth of their social media use are still insufficient. This issue needs the efforts of the whole of society. When helping older adults to use social media, their children or grandchildren share new concepts with the older individuals. Social media managers can build targeted algorithms or guidance strategies to improve the depth and breadth of older adults’ use of social media. The government should also take specific measures and provide policy support to promote the use of social media by older adults.

(2) The findings highlight that attention should be paid to particular older adult groups, and that how it is paid is important. Studies of older people with hearing impairment have revealed the characteristics of this particular group. This group needs more offline attention and interaction than other groups. Therefore, on the one hand, it is necessary to improve the hearing level of older adults with hearing impairment, improve their independent living and offline social ability, and reduce the risk of discrimination. On the other hand, their children, their friends and family, and community workers should not only interact with them on social media, but also increase their targeted offline interaction, such as by accompanying them out and chatting with them at home. More broadly, for particular older adult groups such as those with blindness, physical disability, or cognitive impairment, studies need to explore their attention needs, to improve their physical and mental health.

## 6. Conclusions and Limitations

Based on the S-O-R theory, this study explored the effects of social media use on the subjective aging and mental health of older people with hearing impairment. The study found the following: (1) Social media use has a significant impact on the social relations of hearing-impaired older adults. (2) Social support and social isolation have a significant impact on the psychosocial loss of this group of persons 60 years and older, but it has been proven that the impact of social network size is not established. (3) The self-efficacy of the hearing-impaired older adults was affected by social media use and social support, of which social support was the more influential. (4) Compared with self-efficacy, social isolation and psychosocial loss have a greater impact on depressive symptoms in older people with hearing impairment. The results show that social media use can promote the interaction of strong social relationships among hearing-impaired older adults and has a positive effect on improving their aging cognition and depressive symptoms, but its use is not as good as expected in improving the interaction of weak social relationships. Affected by the decline of their communication abilities, older people with hearing impairment are more eager for offline social interaction. Strengthening social interaction is of great significance to the mental health of older individuals. This study helps to expand the theories of social media, subjective aging, social support, and social networks, and can provide practical contributions to social media use and the mental health of special groups of persons 60 years and older.

This study discusses the mental health problems of older people with hearing impairment and draws some valuable conclusions, but there are also some deficiencies. First, depressive symptoms in older adults are a complex issue, and the study focused on the effects of social media use without considering other factors, such as other chronic diseases, marital status, intergenerational relationships, living environments, or pre-retirement occupations. At the same time, older people who do not use social media are not considered; therefore, the relationship between social relations, subjective aging, and the mental health of older people cannot be comprehensively discussed. Therefore, more influential factors and groups should be considered in the future, in order to explore the causes of depressive symptoms in this group of older people more comprehensively. Second, this study adopts the method of cross-sectional investigation. This means the results of this study are unable to reflect the long-term effects of social media use on older people with hearing impairment; it is also impossible to clearly judge the cause-and-effect relationship between hearing impairment and depressive symptoms. Therefore, future longitudinal research and experimental research are needed to fully explore the impact of social media use. Finally, our research objects are only hearing-impaired older adults living in China, and the vast majority of them use WeChat, a single type of social media. The impact of social media may differ in different regions. Therefore, future research may focus on the effects of different countries, different cultural backgrounds, and different types of social media use on subjective aging and mental health.

## Figures and Tables

**Table 1:** Variables and indicators.

Latent Variable	Measurement Item	Reference
Social Media Use	SMU1: How long have you been using social media (years)?	[71]
	SMU2: How much time per week do you spend on social media?	
	SMU3: How many times per day do you respond to messages, photos, or videos of others on social media?	
	SMU4: How many times per day do you send a message, photo, or video via your social media?	
Social Network Size	SNS1: I have many family members to keep in touch with.	[72]
	SNS2: I have many friends to keep in touch with.	
	SNS3: I have many good acquaintances to keep in touch with.	
	SNS4: I maintain a good relationship with my neighbors.	
Social Isolation	SI1: Overall, I feel that my relationships are fulfilling.	[74]
	SI2: I feel like I just do not belong.	
	SI3: I feel that I spend enough time involved in social activities.	
Social Support	SS1: Someone to give you good advice about a crisis.	[73]
	SS2: Someone to help with daily chores if you were sick.	
	SS3: Someone to confide in or talk to about yourself or your problems.	
	SS4: Someone to show you love and affection.	
	SS5: Someone to have a good time with.	
Psychosocial Loss	PL1: I do not feel involved in society now that I am older.	[75]
	PL2: As I get older, I find it more difficult to make new friends.	
	PL3: I feel excluded from things because of my age.	
	PL4: I find it more difficult to talk about my feelings as I get older.	
	PL5: Old age is a depressing time of life.	
	PL6: Old age is a time of loneliness.	
Self-Efficacy	SE1: I can always manage to solve difficult problems if I try hard enough.	[76]
	SE2: If someone opposes me, I can find the means and ways to get what I want.	
	SE3: It is easy for me to stick to my aims and accomplish my goals.	
	SE4: I am confident that I could deal efficiently with unexpected events.	
	SE5: Thanks to my resourcefulness, I know how to handle unforeseen situations.	
	SE6: I can solve most problems if I invest the necessary effort.	
	SE7: I can remain calm when facing difficulties because I can rely on my coping skills.	
	SE8: When I am confronted with a problem, I can usually find several solutions.	
	SE9: If I am in trouble, I can usually think of a solution.	
	SE10: I can usually handle whatever comes my way.	
Depressive Symptoms	DS1: Are you basically satisfied with your life?	[77]
	DS2: Do you often get bored?	
	DS3: Do you often feel helpless?	
	DS4: Do you prefer to stay at home rather than going out and doing new things?	
	DS5: Do you feel worthless the way you are now?	

**Table 2:** Sample characteristics.

Measure	Item	Count	Measure	Item	Count
Gender	Male	316 (49.14%)	Hearing Loss	One ear	36 (5.60%)
	Female	327 (50.86%)		Both ears	607 (94.40%)
Age	60–64	294 (45.72%)	Self-Reported Hearing Disability	Very easy	266 (41.37%)
	65–69	187 (29.08%)		Fairly easy	171 (26.50%)
	70–74	102 (15.86%)		Fairly hard	143 (22.23%)
	75–79	39 (6.02%)		Very hard	63 (9.90%)
	>80	21 (3.32%)		None	398 (61.90%)
Income	<2000 (~USD300)	173 (26.90%)	Hearing Devices	Cochlear implants	15 (2.33%)
	2000–4000 (~USD300–600)	203 (31.57%)		Bone-anchored hearing aids	21 (3.27%)
	4000–6000 (~USD600–900)	186 (28.93%)		Air-conduction hearing aids	177 (27.52%)
	>6000 (~USD900)	81 (12.60%)		Other	32 (4.98%)

**Table 3:** Data descriptive statistics results.

Construct	Item	Mean	SD
Social Isolation	SI1	4.918	1.596
	SI2	4.792	1.540
	SI3	5.056	1.602
Psychosocial Loss	PL1	4.900	1.601
	PL2	4.899	1.538
	PL3	4.844	1.613
	PL4	4.975	1.646
	PL5	4.846	1.515
	PL6	4.967	1.634
Self-Efficacy	SE1	5.003	1.554
	SE2	4.928	1.584
	SE3	5.008	1.516
	SE4	5.042	1.508
	SE5	4.997	1.506
	SE6	4.997	1.520
	SE7	5.033	1.582
	SE8	5.036	1.584
	SE9	4.942	1.590
	SE10	4.952	1.476
Social Network Size	SNS1	5.025	1.590
	SNS2	4.935	1.600
	SNS3	4.988	1.678
	SNS4	4.843	1.680
Social Support	SS1	4.866	1.508
	SS2	4.827	1.563
	SS3	4.759	1.633
	SS4	5.002	1.583
	SS5	4.966	1.564
Social Media Use	SMU1	4.918	1.613
	SMU2	4.946	1.616
	SMU3	4.751	1.517
	SMU4	4.988	1.523
Depressive Symptoms	DS1	4.986	1.591
	DS2	4.894	1.527
	DS3	4.946	1.675
	DS4	4.933	1.639
	DS5	4.863	1.581

**Table 4:** Reliability and validity.

Construct	Item	Loading	Cronbach’s Alpha	Rho_A	CR	AVE
Social Isolation	SI1	0.826	0.765	0.770	0.865	0.680
	SI2	0.800				
	SI3	0.848				
Psychosocial Loss	PL1	0.765	0.855	0.857	0.892	0.579
	PL2	0.766				
	PL3	0.756				
	PL4	0.760				
	PL5	0.759				
	PL6	0.758				
Self-Efficacy	SE1	0.730	0.900	0.922	0.916	0.523
	SE2	0.764				
	SE3	0.726				
	SE4	0.709				
	SE5	0.703				
	SE6	0.720				
	SE7	0.712				
	SE8	0.727				
	SE9	0.714				
	SE10	0.735				
Social Network Size	SNS1	0.763	0.822	0.859	0.880	0.648
	SNS2	0.853				
	SNS3	0.802				
	SNS4	0.800				
Social Support	SS1	0.781	0.830	0.831	0.880	0.595
	SS2	0.766				
	SS3	0.763				
	SS4	0.786				
	SS5	0.763				
Social Media Use	SMU1	0.783	0.824	0.886	0.879	0.644
	SMU2	0.773				
	SMU3	0.859				
	SMU4	0.793				
Depressive Symptoms	DS1	0.785	0.839	0.841	0.886	0.609
	DS2	0.733				
	DS3	0.816				
	DS4	0.790				
	DS5	0.774				

**Table 5:** Results of the discriminant validity analysis.

	DS	PL	SE	SI	SMU	SNS	SS
DS	0.780						
PL	0.406	0.761					
SE	0.188	0.112	0.723				
SI	0.411	0.483	0.199	0.825			
SMU	0.125	0.142	0.118	0.107	0.803		
SNS	0.181	0.138	0.12	0.188	0.132	0.805	
SS	0.088	0.222	0.186	0.254	0.129	0.437	0.772

Notes: DS = Depressive Symptoms; PL = Psychosocial Loss; SE = Self-Efficacy; SI = Social Isolation; SMU = Social Media Use; SNS = Social Network Size; SS = Social Support.

**Table 6:** Hypothesis testing.

Hypothetical Path	Path Coefficient	T Value	*p* Value	Conclusion
H1 SMU -> SNS	0.132	3.444	0.001	Support
H2 SMU -> SS	0.129	2.950	0.003	Support
H3 SMU -> SI	0.107	2.505	0.013	Support
H4 SNS -> PL	0.007	0.182	0.856	No support
H5 SS -> PL	0.103	2.014	0.044	Support
H6 SI -> PL	0.456	10.458	<0.001	Support
H7 SMU -> SE	0.096	2.249	0.025	Support
H8 SS -> SE	0.174	4.434	<0.001	Support
H9 PL -> DS	0.268	6.307	<0.001	Support
H10 SE -> DS	0.106	3.150	0.002	Support
H11 SI -> DS	0.260	6.036	<0.001	Support

Notes: DS = Depressive Symptoms; PL = Psychosocial Loss; SE = Self-Efficacy; SI = Social Isolation; SMU = Social Media Use; SNS = Social Network Size; SS = Social Support.

## Data Availability

Not applicable.
